# Supplementary material for: Identification of DNA methylation-regulated genes as potential biomarkers for coronary heart disease via machine learning in the Framingham Heart Study
Source: Clin Epigenetics. 2022 Sep 30;14:122. doi: 10.1186/s13148-022-01343-2 (PMC9526342; doi:10.1186/s13148-022-01343-2)
Supplement: Supplementary file 1 — Additional file 1: Table S1. Performance of the expression and methylation models applied to datasets from GEO. Table S2. Clinical baseline information of participants in the transcriptome sequencing and methylation microarray. [file 13148_2022_1343_MOESM1_ESM.docx]

**Table S1.** Performance of the expression and methylation models applied to datasets from GEO.

| **Features** | **Algorithm** | **Dataset** | **F1** | **ACC** | **AUC (95% CI)** | **AP** | **KS** | **TP** | **FP** | **TN** | **FN** | **TPR** | **TNR** | **Kappa** |
| --- | --- | --- | --- | --- | --- | --- | --- | --- | --- | --- | --- | --- | --- | --- |
| Methylation | LightGBM | GSE107143 | 0.667 | 0.688 | 0.672 (0.358–0.985) | 0.600 | 0.500 | 5 | 2 | 6 | 3 | 0.625 | 0.750 | 0.375 |
| Expression | LightGBM | GSE42148 | 0.667 | 0.667 | 0.699 (0.501–0.897) | 0.733 | 0.434 | 8 | 3 | 8 | 5 | 0.615 | 0.727 | 0.338 |

ACC, accuracy; AUC, area under the receiver operating characteristic curve; CI, confidence interval; AP, average precision score; KS, Kolmogorov-Smirnov; TP, true positive; FP, false positive; TN, true negative; FN, false negative; TPR, true positive rate; TNR, true negative rate.

**Table S2.** Clinical baseline information of participants in the transcriptome sequencing and methylation microarray.

| **Characteristic** | **Controls** | **CHD patients** | ***P* value** |
| --- | --- | --- | --- |
| Male / Female | 12 / 0 | 12 / 0 | / |
| Age (year) | 60.75 ± 3.39 | 60.67 ± 3.75 | 0.9550 ^a^ |
| WBC (10^9^/L) | 5.28 ± 0.78 | 6.30 ± 1.78 | 0.0792 ^a^ |
| Monocyte (%) | 7.05 (6.75, 7.80) | 7.80 (7.20, 8.55) | 0.1640 ^b^ |
| LDL-C (mmol/L) | 2.27 ± 0.29 | 2.69 ± 0.88 | 0.1318 ^a^ |
| HDL-C (mmol/L) | 1.29 (1.13, 1.58) | 1.02 (0.91, 1.21) | 0.0138 ^b^ |
| TG (mmol/L) | 1.02 ± 0.32 | 1.51 ± 0.69 | 0.0390 ^a^ |

Data were showed as mean ± SD or median (interquartile range).WBC, white blood cell; LDL-C, low-density lipoprotein cholesterol; HDL-C, high-density lipoprotein cholesterol; TG, triglycerides. ^a^ Student’s *t* test. ^b^ Mann-Whitney *U* test.
